# Morphine Antinociception Restored by Use of Methadone in the Morphine-Resistant Inflammatory Pain State

**DOI:** 10.3389/fphar.2020.593647

**Published:** 2020-12-04

**Authors:** Chizuko Watanabe, Asami Komiyama, Masaru Yoshizumi, Shinobu Sakurada, Hirokazu Mizoguchi

**Affiliations:** Department of Physiology and Anatomy, Faculty of Pharmaceutical Sciences, Tohoku Medical and Pharmaceutical University, Sendai, Japan

**Keywords:** antinociception, methadone, morphine, inflammatory pain, restore

## Abstract

The antinociceptive effect of methadone in the morphine-resistant inflammatory pain state was described in the paw-withdrawal test using the complete Freund’s adjuvant (CFA)-induced mouse inflammatory pain model. After intraplantar (i.pl.) injection of CFA, thermal hyperalgesia was observed in the ipsilateral paw. The antinociceptive effects of subcutaneous (s.c.) injection of morphine, fentanyl, and oxycodone against thermal hyperalgesia in the inflammatory pain state were reduced in the ipsilateral paw 7 days after CFA pretreatment. On the contrary, the antinociceptive effect of s.c. injection of methadone was maintained in the ipsilateral paw 7 days after CFA pretreatment. The suppressed morphine antinociception in the CFA model mice was bilaterally restored following s.c. treatment with methadone 20 min prior to or 3 days after CFA pretreatment. The suppressed morphine antinociception was also bilaterally restored by intraperitoneal treatment with MK-801 30 min prior to CFA pretreatment; however, the s.c. injection of morphine 30 min prior to CFA pretreatment failed to restore the suppressed morphine antinociception in the CFA model mice. The expression level of mRNA for µ-opioid receptors 7 days after i.pl. pretreatment was not significantly changed by i.pl. pretreatment with CFA or s.c. pretreatment with methadone. In conclusion, methadone is extremely effective against thermal hyperalgesia in the morphine-resistant inflammatory pain state, and restores suppressed morphine antinociception in the inflammatory pain state without altering the expression level of mRNA for µ-opioid receptors.

## Introduction

Inflammatory pain is the spontaneous hypersensitivity to pain that occurs in response to tissue damage and inflammation. The prominent features of inflammatory pain are edema, mechanical allodynia, thermal hyperalgesia, and mechanical hyperalgesia. Several experimental models of inflammatory pain have been developed in rodents, including formalin- (Hunskaar and Hole, 1987), carrageenan- (Miyake et al., 2019), and complete Freund’s adjuvant (CFA)-based models (Ohsawa et al., 2000). Among the experimental models of inflammatory pain, the inflammatory pain model involving hind-paw inflammation caused by intraplantar (i.pl.) administration of CFA is widely used to describe the mechanisms of inflammatory pain and the effectiveness of analgesics against inflammatory pain. Inflammatory pain in this model is distinct from pain in other chronic pain models where expression is restricted to the ipsilateral side. Mechanical allodynia in the CFA inflammatory pain model is observed bilaterally in both the ipsilateral (inflamed) and contralateral (non-inflamed) paw, whereas thermal hyperalgesia is observed only in the ipsilateral paw (Nagakura et al., 2003). Therefore, the mechanism underlying this inflammatory pain is considered to be more complicated than that underlying other chronic pain.

It is well established that the antinociceptive effect of morphine against both hyperalgesia and allodynia is suppressed in chronic pain, such as neuropathic pain (Narita et al., 2008), neuropathic cancer pain (Luger et al., 2002), and diabetic neuropathic pain (Zurek et al., 2001). Therefore, these types of pain are known as morphine-resistant intractable pain. Unlike morphine-resistant intractable pain, the antinociceptive effect of morphine is reported to be enhanced or retained against mechanical hyperalgesia (Maldonado et al., 1994; Fernández-Dueñas et al., 2007) and thermal hyperalgesia (Fernández-Dueñas et al., 2007) in the CFA-induced inflammatory pain state. However, in a clinical study, morphine was found to be less effective against inflammatory pain (Lillesø et al., 2000). We previously found that the antinociceptive effect of morphine was markedly suppressed against mechanical allodynia in the inflammatory pain state (Aoki et al., 2014b). In the present study, we further characterized the antinociceptive effect of narcotic analgesics, including morphine, against thermal hyperalgesia in the inflammatory pain state, and found a special effect of methadone in restoring suppressed morphine antinociception in the inflammatory pain state.

## Materials and Methods

All experiments were performed following the approval of the Ethics Committee for Animal Experiments at Tohoku Medical and Pharmaceutical University and according to the National Institutes of Health Guide for the Care and Use of Laboratory Animals. Every effort was made to minimize the number and suffering of the animals used in the following experiments.

### Animals

Male ddY mice (Japan SLC, Hamamatsu, Japan) weighing 18–24 g were used. Mice were housed in a temperature- (22–23°C) and humidity-controlled (50–60%) room with an alternating 12-h light/dark (lights on at 07:00 and off at 19:00) cycle. Food and water were available ad libitum.

### Mouse Model for Inflammatory Pain

To produce the inflammatory pain model, mice underwent i.pl. injection of 50 µL of CFA (Sigma-Aldrich, St. Louis, MO) in the left hind paw using a syringe with a 26-gauge needle (Aoki et al., 2014a; Aoki et al., 2014b). Control mice underwent i.pl. injection of sterile saline.

### Measurement of Thermal Hyperalgesia and Antinociception

The thermal hyperalgesia and antinociceptive effect of narcotic analgesics were measured using the paw-withdrawal test, using an automated tail-flick unit (Ugo Basile, Italy) (Mizoguchi et al., 2006). Mice were adapted to the testing environment for at least 1 h before stimulation. Each animal was restrained with a soft cloth to reduce visual stimulation, and a light beam was applied to the hind paw as a noxious radiant heat stimulus. The light beam focused on the plantar surface of the hind paw, and the latency of the paw withdrawal in response to noxious radiant heat stimulation was measured. The intensity of the noxious radiant heat stimulation was adjusted such that the pre-latency for the paw withdrawal response was approximately 6 s. The antinociceptive effect was expressed as a percentage of the maximum possible effect (% MPE), which was calculated using the following equation: ([T1 − T0]/[10 − T0]) × 100, where T0 and T1 are the pre- and post-drug latencies for the paw withdrawal response, respectively. To prevent tissue damage of the paw, noxious radiant heat stimulation was terminated automatically if the mouse did not lift the paw within 10 s.

### Drugs

The drugs used were morphine hydrochloride (Takeda, Osaka, Japan), fentanyl citrate (Mallinckrodt Pharmaceuticals, St. Louis, MO), oxycodone hydrochloride (Mallinckrodt Pharmaceuticals), methadone hydrochloride (Mallinckrodt Pharmaceuticals), and (5*R*,10*S*)-(+)-5-methyl-10,11-dihydro-5H-dibenzo[*a*,*d*]cycloheptene-5,10-imine (MK-801) hydrogen maleate (Sigma-Aldrich Chemical Co.). All drugs were dissolved in sterile saline.

### Semi-Quantitative Reverse Transcription-Polymerase Chain Reaction

Total RNA in the mouse lumbar spinal cord was extracted using the RNeasy Lipid Tissue Mini Kit (QIAGEN K.K., Tokyo, Japan). The purified total RNA was quantified using a spectrophotometer at A260. RT-PCR amplification was performed using the SuperScript One Step RT-PCR system Platinum version (Life Technologies, Carlsbad, CA) (Aoki et al., 2014b). The synthetic forward and reverse primers for the µ-opioid receptor were 5’-CAG CCA GCA TTC AGA ACC ATG G-3’ and 5’-ATG GTG CAG AGG GTG AAG ATA CTG G-3’, respectively. The synthetic forward and reverse primers for β-actin were 5’-GCT CGT CGT CGA CAA CGG CTC-3’ and 5’-CAA ACA TGA TCT GGG TCA TCT TCT T-3’, respectively. The samples were heated to 50°C for 30 min for cDNA synthesis and to 94°C for 2 min for pre-denaturation, and then cycled 30 times through 94°C for 30 s for denaturation, 55°C for 30 s for annealing, and 72°C for 30 s for extension, and finally heated to 72°C for 7 min for final extension. The resulting PCR product was electrophoresed on a 0.8% agarose gel containing ethidium bromide. The agarose gel was photographed using a UV Imaging Analyzer FAS-III (Toyobo, Osaka, Japan); the PCR product for the µ-opioid receptor in agarose gel was then semi-quantified in relation to β-actin using a Lumino Imaging Analyzes FAS-1000 (Toyobo).

### Statistical Analysis

The data are expressed as the mean ± SEM for at least eight mice. The statistical significance of differences between the groups was assessed with a one-way analysis of variance (ANOVA) or repeated measures two-way ANOVA followed by Bonferroni’s test.

## Results

### CFA-Induced Thermal Hyperalgesia

**FIGURE 1:**
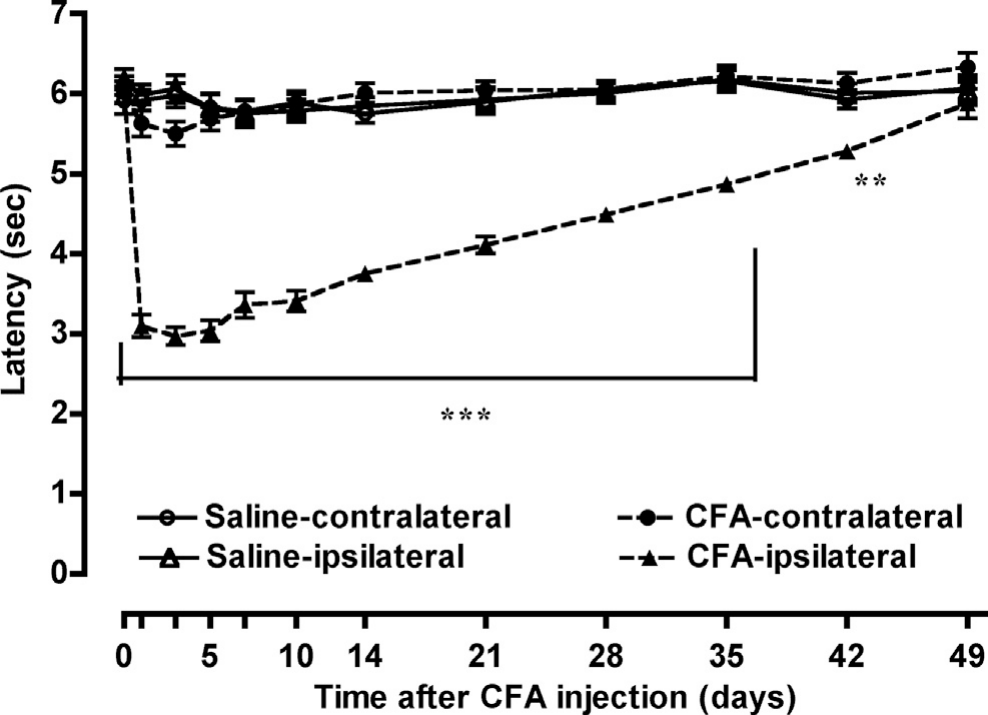
CFA-induced thermal hyperalgesia. Groups of mice were injected i.pl. with 50 µL of saline or CFA in the left hind paw; the withdrawal threshold in the ipsilateral and contralateral paws in response to thermal stimulation of the plantar surface of each hind paw was measured for 49 days. Each value represents the mean ± S.E.M. for 12–14 mice. **p < 0.01, ***p < 0.001 vs. ipsilateral paw on the control model (i.pl. saline pretreatment) mice.

Groups of mice were injected i.pl. with 50 µL of saline or CFA in the left hind paw, and the withdrawal threshold in response to thermal stimulation of the plantar surface of each hind paw was measured for 49 days. After CFA injection, a decrease in the thermal withdrawal threshold (thermal hyperalgesia) was observed in the ipsilateral paw, but not the contralateral paw (Figure 1). Thermal hyperalgesia in the ipsilateral paw of the CFA-treated mice peaked 5 days after CFA treatment and was maintained for 42 days after CFA treatment. In contrast, the thermal withdrawal threshold was not altered in mice that received i.pl. saline treatment.

### Antinociceptive Effect of Narcotic Analgesics in the Inflammatory Pain State

**FIGURE 2:**
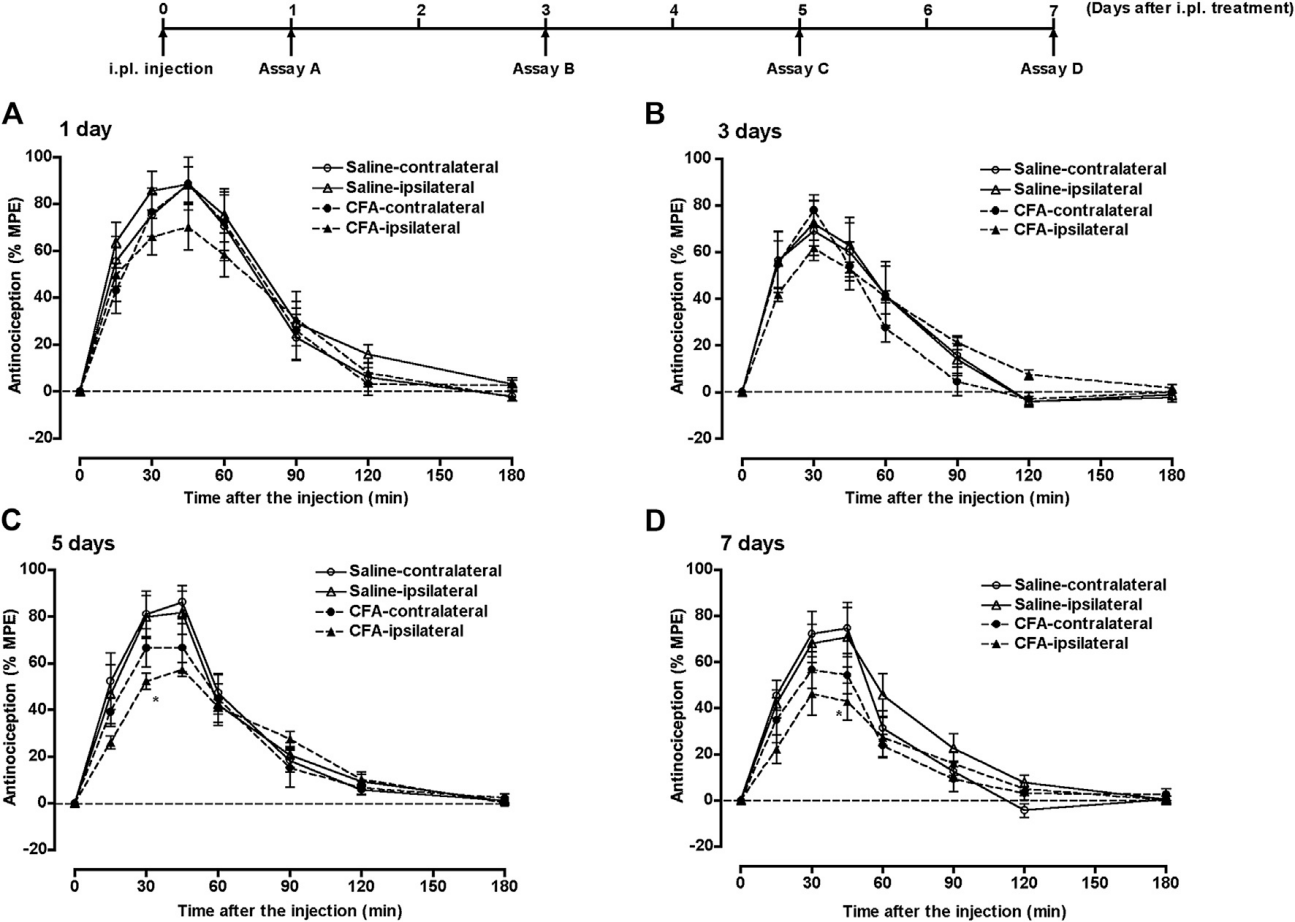
The antinociceptive effect of morphine in the inflammatory pain state. Groups of mice pretreated i.pl. with 50 µL of saline or CFA in the left hind paw were injected s.c. with morphine (5 mg/kg) at 1 **(A)**, 3 **(B)**, 5 **(C)** or 7 days **(D)** after i.pl. pretreatment; the withdrawal threshold in response to thermal stimulation of the plantar surface of each hind paw was measured for 180 min. Each value represents the mean ± S.E.M. for eight mice. *p < 0.05 vs. ipsilateral paw in the control model (i.pl. saline pretreatment) mice.

Groups of mice pretreated with i.pl. 50 µL of saline or CFA in the left hind paw, underwent subcutaneous (s.c.) injection of morphine (5 mg/kg) at 1, 3, 5, or 7 days after the i.pl. pretreatment, and the withdrawal threshold against thermal stimulation to the plantar surface of each hind paw was measured for 180 min. The potent increase in withdrawal threshold (antinociceptive effect) following morphine treatment was observed bilaterally in saline-pretreated mice at all time points (Figure 2). However, the antinociceptive effect of morphine in the ipsilateral paw was gradually suppressed after CFA pretreatment, and was significantly suppressed at 5 and 7 days after CFA treatment (Figures 2C,D). The antinociceptive effect of morphine in the contralateral paw also tended to be suppressed at 5 and 7 days after CFA treatment.

**FIGURE 3:**
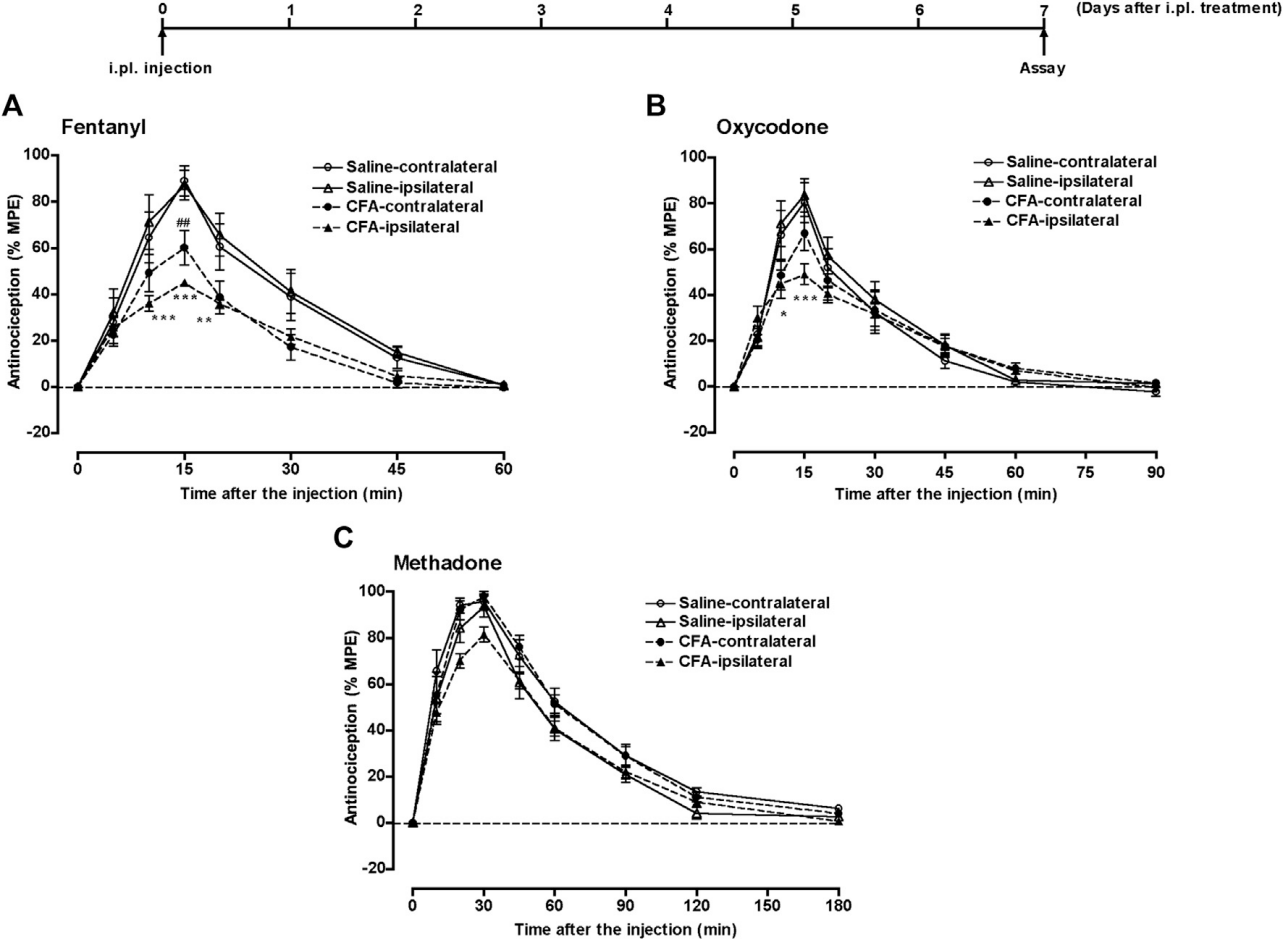
The antinociceptive effect of narcotic analgesics in the inflammatory pain state. Groups of mice pretreated i.pl. with 50 µL of saline or CFA in the left hind paw, were injected s.c. with fentanyl **(A)** (0.08 mg/kg), oxycodone **(B)** (1.5 mg/kg), or methadone **(C)** (2.8 mg/kg) at 7 days after i.pl. pretreatment; the withdrawal threshold in response to thermal stimulation of the plantar surface of each hind paw was measured for 60, 90, or 180 min, respectively. Each value represents the mean ± S.E.M. for eight mice. *p < 0.05, **p < 0.01, ***p < 0.001 vs. ipsilateral paw in the control model (i.pl. saline pretreatment) mice. ##*p* < 0.01 vs. contralateral paw in the control model (i.pl. saline pretreatment) mice.

Another group of mice that underwent i.pl. pretreatment with 50 µL of saline or CFA in the left hind paw, underwent s.c. injection of fentanyl (0.08 mg/kg), oxycodone (1.5 mg/kg), or methadone (2.8 mg/kg) 7 days after i.pl. pretreatment; the withdrawal threshold in response to thermal stimulation of the plantar surface of each hind paw was measured for 60, 90, or 180 min, respectively. The potent antinociceptive effects of these narcotic analgesics was observed bilaterally in saline-pretreated mice (Figure 3). However, the antinociceptive effect of fentanyl was significantly suppressed bilaterally 7 days after CFA pretreatment (Figure 3A). The antinociceptive effect of oxycodone was significantly suppressed 7 days after CFA pretreatment in the ipsilateral paw, but not in the contralateral paw (Figure 3B). In contrast, the antinociceptive effects of methadone in both paws was not affected 7 days after CFA treatment (Figure 3C).

### Recovery of Antinociceptive Effect of Morphine in Inflammatory Pain State

**FIGURE 4:**
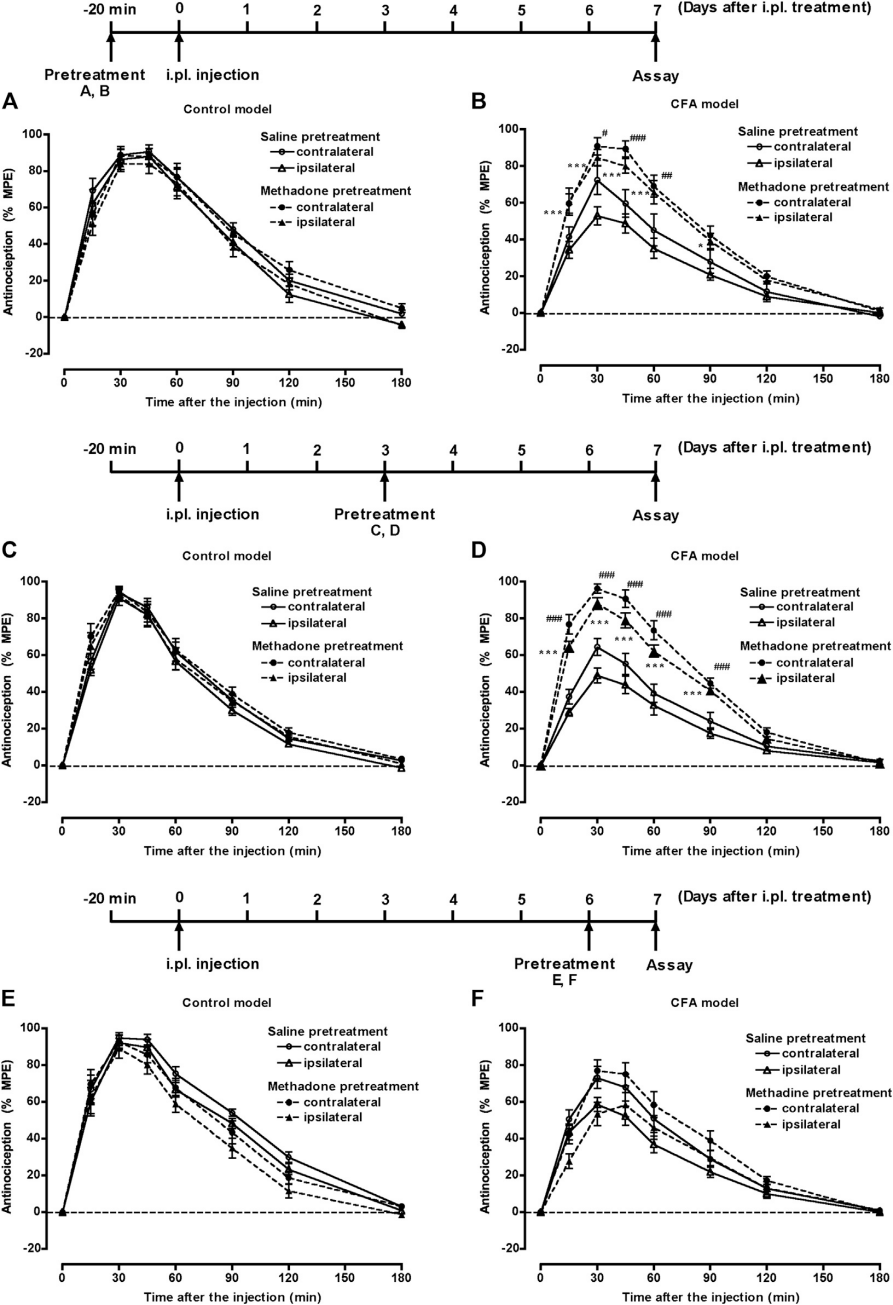
The effect of methadone on suppressed morphine antinociception in the inflammatory pain state. Groups of mice pretreated i.pl. with 50 µL of saline **(A, C, E)** or CFA **(B, D, F)** in the left hind paw, were injected s.c. with saline or methadone (11.2 mg/kg) 20 min prior to **(A, B)**, 3 days after **(C, D)**, or 6 days after **(E, F)** i.pl. pretreatment. Seven days after i.pl. pretreatment, mice were injected s.c. with morphine (5 mg/kg), and the withdrawal threshold in response to thermal stimulation of the plantar surface of each hind paw was measured for 180 min. Each value represents the mean ± S.E.M. for eight mice. *p < 0.05, ***p < 0.001 vs. ipsilateral paw in the s.c. saline-pretreated mice. #*p* < 0.05, ##*p* < 0.01, ###*p* < 0.001 vs. contralateral paw in the s.c. saline-pretreated mice.

Groups of mice pretreated with 50 µL of saline or CFA i.pl. in the left hind paw, were injected s.c. with saline or methadone (11.2 mg/kg) 20 min prior, 3 days after, or 6 days after i.pl. pretreatment. Seven days after the i.pl. pretreatment, mice were injected s.c. with morphine (5 mg/kg), and the withdrawal threshold against thermal stimulation of the plantar surface of each hind paw was measured for 180 min. In the control model (i.pl. saline pretreatment) mice, morphine showed a bilateral potent antinociceptive effect with any s.c. pretreatment with saline or methadone (Figures 4A,C,E). In the CFA model (i.pl. CFA pretreatment) mice, the antinociceptive effect of morphine was bilaterally suppressed. However, the suppressed morphine antinociception in the CFA model mice was bilaterally restored following s.c. treatment with methadone 20 min prior to or 3 days after CFA pretreatment (Figures 4B,D). On the contrary, s.c. treatment with methadone 6 days after CFA pretreatment failed to restore the suppressed morphine antinociception in the CFA model mice (Figure 4F).

**FIGURE 5:**
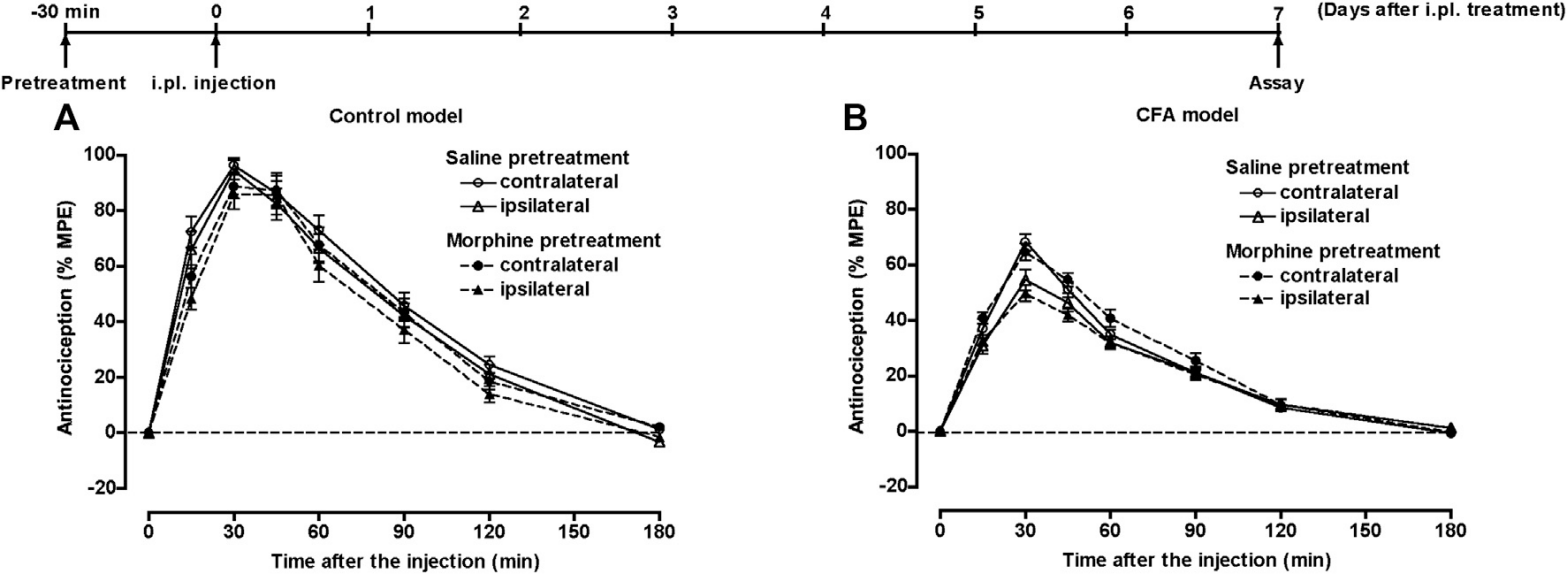
The effect of morphine on suppressed morphine antinociception in the inflammatory pain state. Groups of mice pretreated i.pl. with 50 µL of saline **(A)** or CFA **(B)** in the left hind paw were injected s.c. with saline or morphine (20 mg/kg) 30 min prior to i.pl. pretreatment. Seven days after i.pl. pretreatment, mice were injected s.c. with morphine (5 mg/kg), and the withdrawal threshold in response to thermal stimulation of the plantar surface of each hind paw was measured for 180 min. Each value represents the mean ± S.E.M. for eight mice.

**FIGURE 6:**
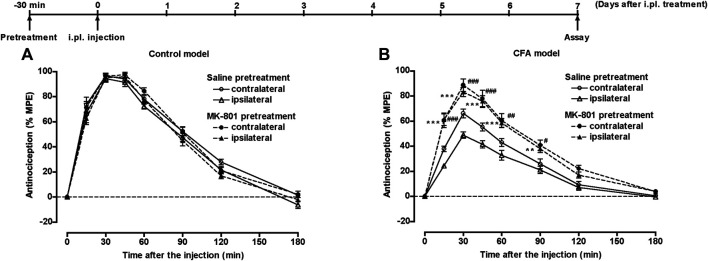
The effect of MK-801 on suppressed morphine antinociception in the inflammatory pain state. Groups of mice pretreated i.pl. with 50 µL of saline **(A)** or CFA **(B)** in the left hind paw, were injected i.p. with saline or MK-801 (0.25 mg/kg) 30 min prior to i.pl. pretreatment. Seven days after i.pl. pretreatment, mice were injected s.c. with morphine (5 mg/kg), and the withdrawal threshold in response to thermal stimulation of the plantar surface of each hind paw was measured for 180 min. Each value represents the mean ± S.E.M. for eight mice. **p < 0.01, ***p < 0.001 vs. ipsilateral paw in s.c. saline-pretreated mice. #*p* < 0.05, ##*p* < 0.01, ###*p* < 0.001 vs. contralateral paw in s.c. saline-pretreated mice.

Another group of mice pretreated with 50 µL of saline or CFA i.pl. in the left hind paw, underwent s.c. injection of saline or morphine (20 mg/kg) or intraperitoneal (i.p.) injection of saline or MK-801 (0.25 mg/kg) 30 min prior to i.pl. pretreatment. Seven days after the i.pl. pretreatment, mice underwent s.c. injection of morphine (5 mg/kg), and the withdrawal threshold in response to thermal stimulation of the plantar surface of each hind paw was measured for 180 min. In control mice, morphine bilaterally showed a potent antinociceptive effect following s.c. pretreatment with saline or morphine (Figure 5A) or i.p. pretreatment with saline or MK-801 (Figure 6A). In the CFA model mice, the antinociceptive effect of morphine was bilaterally suppressed. The suppressed morphine antinociception was bilaterally restored following i.p. treatment with MK-801 30 min prior to CFA pretreatment (Figure 6B); however, s.c. treatment with morphine 30 min prior to CFA pretreatment failed to restore the suppressed morphine antinociception in the CFA model mice (Figure 5B).

### mRNA Expression of µ-Opioid Receptors in the Inflammatory Pain State

**FIGURE 7:**
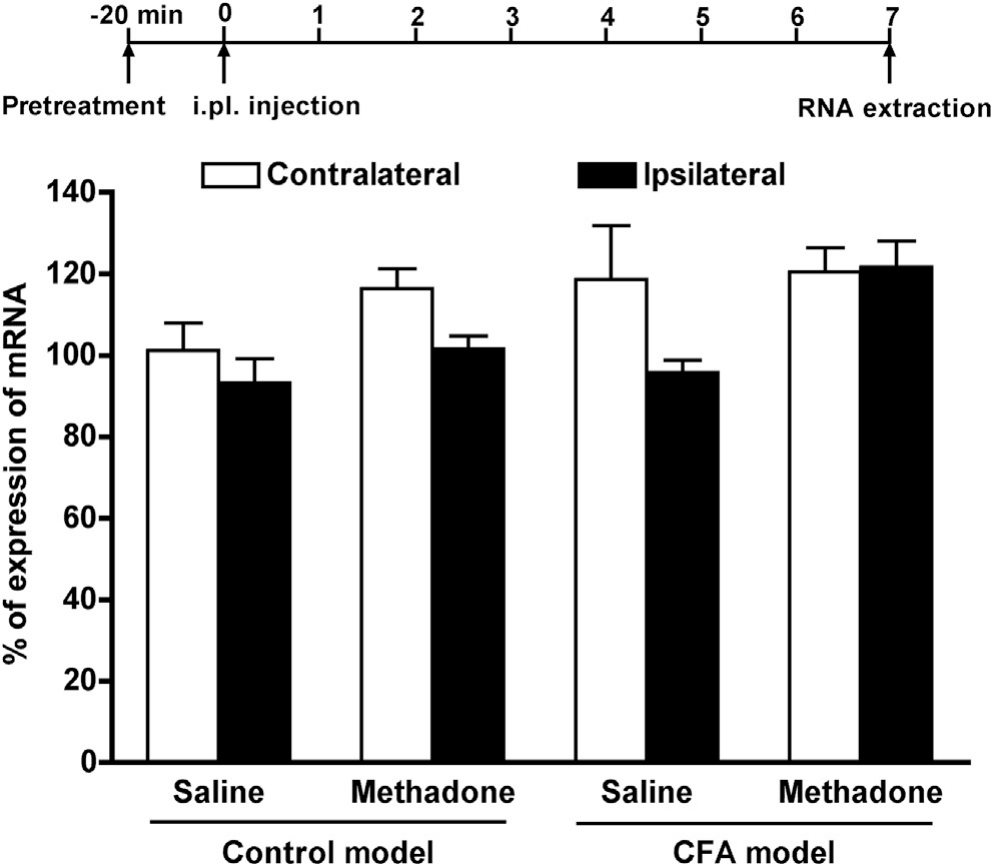
The expression level of mRNA for µ-opioid receptors in lumbar spinal cord in the inflammatory pain state. Groups of control model mice and CFA model mice were injected s.c. with saline or methadone (11.2 mg/kg), and the expression level of mRNA for µ-opioid receptors in the lumbar spinal cord on the ipsilateral side and contralateral side 7 days after CFA pretreatment was measured using semi-quantitative RT-PCR. Data are expressed as the % of the mRNA level for µ-opioid receptors normalized to that of β-actin; each value represents the mean ± S.E.M. for duplicated three independent sets of experiments.

The expression level of mRNA for µ-opioid receptors in the lumbar spinal cord on the ipsilateral and contralateral sides on the inflammatory pain was measured using semi-quantitative RT-PCR. The total RNA in the lumbar spinal cord of the ipsilateral side and contralateral side was extracted 7 days after CFA pretreatment, and the expression level of mRNA for µ-opioid receptors was measured using semi-quantitative RT-PCR. The expression level of mRNA for µ-opioid receptors in the lumbar spinal cord was not bilaterally altered by CFA pretreatment (Figure 7). Moreover, pretreatment with methadone did not bilaterally alter the expression level of µ-opioid receptor mRNA in either the control or CFA models.

## Discussion

We previously determined the antinociceptive effect of morphine against mechanical allodynia in the CFA-induced inflammatory pain state in mice using the von Frey filament test (Aoki et al., 2014b). In that study, the antinociceptive effect of morphine against mechanical allodynia was bilaterally suppressed at 1 and 4 days after CFA treatment. However, in the present study using the same inflammatory pain model, the antinociceptive effect of morphine against thermal hyperalgesia was not suppressed at 1 and 3 days after CFA treatment, and was significantly suppressed in the ipsilateral paw at 5 and 7 days after CFA treatment. Both mechanical allodynia and thermal hyperalgesia in the CFA-induced inflammatory pain state were quickly observed 1 day after CFA treatment and prolonged for approximately 40 days. This evidence clearly suggests that the antinociceptive mechanism of morphine against thermal hyperalgesia is different from that against mechanical allodynia, and both antinociceptive mechanisms are altered by CFA treatment at different times. This may be because there are controversial reports regarding the effectiveness of morphine in the inflammatory pain state (Maldonado et al., 1994; Fernández-Dueñas et al., 2007; Aoki et al., 2014b).

In the present study, the antinociceptive effect of other narcotic analgesics, fentanyl and oxycodone, against thermal hyperalgesia was significantly suppressed in the ipsilateral paw 7 days after CFA treatment. However, the antinociceptive effect of methadone against thermal hyperalgesia was not affected 7 days after CFA treatment. We previously reported that the antinociceptive effect of fentanyl and oxycodone against mechanical allodynia was bilateraly suppressed 1 day after CFA treatment, whereas the antinociceptive effect of methadone against mechanical allodynia was suppressed only in the ipsilateral paw 1 day after CFA treatment (Aoki et al., 2014a). This evidence clearly suggests that among narcotic analgesics, methadone is relatively effective in the CFA-induced inflammatory pain state. Methadone is composed of *l*-methadone, which has agonistic activity against µ-opioid receptors and *d*-methadone, which has antagonistic activity against NMDA receptors (Inturrisi, 2005). The antagonistic activity of *d*-methadone against NMDA receptors may be involved in the retained antinociceptive effect of methadone against thermal hyperalgesia in the CFA-induced inflammatory pain state. However, in the present study, co-administration of fentanyl (0.08 mg/kg, s.c.) and MK-801 (0.25 mg/kg, i.p.), an NMDA receptor antagonist, failed to potentiate the antinociceptive effect of fentanyl against thermal hyperalgesia in the CFA-induced inflammatory pain state (data not shown). Therefore, the retained antinociceptive effect of methadone against thermal hyperalgesia in the CFA-induced inflammatory pain state may be mediated through a mechanism not related to the antagonism of NMDA receptors.

The most fundamental finding in the present study is that a single treatment of methadone restored the suppressed antinociceptive effect of morphine against thermal hyperalgesia in the CFA-induced inflammatory pain state. The suppressed antinociceptive effect of morphine against thermal hyperalgesia in the CFA-induced inflammatory pain state was completely restored by a single treatment with a high dose of methadone (four times higher dose of 90% antinociception) 20 min prior to or 3 days after, but not 6 days after, CFA pretreatment. The evidence suggests that a single treatment of high-dose methadone in the early phase of inflammation restores the suppressed morphine antinociception against thermal hyperalgesia in the CFA-induced inflammatory pain state. To investigate the mechanism of how methadone restored the suppressed morphine antinociception, the effect of a single treatment with morphine (20 mg/kg, s.c.) or MK-801 (0.25 mg/kg, i.p.) 30 min prior to CFA treatment on suppressed morphine antinociception against thermal hyperalgesia in the CFA-induced inflammatory pain state was examined. As a result, a single treatment with MK-801, but not morphine, restored the suppressed morphine antinociception against thermal hyperalgesia in the CFA-induced inflammatory pain state. The blockade of NMDA receptors by methadone in the early phase of inflammation may be involved in the restoration of suppressed morphine antinociception against thermal hyperalgesia in the CFA-induced inflammatory pain state.

We previously reported that suppressed morphine antinociception against mechanical allodynia in the ipsilateral paw in the early phase of CFA-induced inflammation (1 day after CFA treatment) is reflected in the reduced expression of the µ-opioid receptor mRNA in the ipsilateral dorsal root ganglion and ipsilateral lumbar spinal cord (Aoki et al., 2014b). Therefore, in the present study, the expression level of µ-opioid receptor mRNA was measured 7 days after CFA treatment. As a result, 7 days after CFA treatment, the expression level of µ-opioid receptor mRNA in the ipsilateral lumbar spinal cord was not significantly altered by CFA treatment. Moreover, the expression level of µ-opioid receptor mRNA was not altered by a single pretreatment with methadone. The evidence suggests that the expression of µ-opioid receptor mRNA in the ipsilateral lumbar spinal cord, which was once reduced in the early phase of CFA-induced inflammation, returns to normal levels 7 days after CFA treatment. A single injection of methadone may restore the suppressed morphine antinociception against thermal hyperalgesia in the CFA-induced inflammatory pain state without altering the expression level of µ-opioid receptor mRNA in the ipsilateral lumbar spinal cord.

It has been well established that the phosphorylation of µ-opioid receptors by protein kinase C (PKC) enhances the desensitization of µ-opioid receptors (Bohn et al., 2004; Bailey et al., 2006). We previously identified that in addition to the reduced expression of µ-opioid receptor mRNA, the phosphorylation of µ-opioid receptors by PKC is also involved in the suppression of morphine antinociception against mechanical allodynia in the early phase of CFA-induced inflammation (Aoki et al., 2014b). In the chronic pain state, the activity of PKC in the dorsal spinal cord is enhanced by the activation of NMDA receptors in postsynaptic cells (Mao et al., 1995a; Mao et al., 1995b). Therefore, the activation of NMDA receptors causes the phosphorylation of co-localized µ-opioid receptors, which results in the desensitization of µ-opioid receptors (Sánchez-Blázquez et al., 2013). In fact, the development of hyperalgesia and allodynia in morphine-resistant chronic pain states is suppressed by NMDA receptor antagonists or PKC inhibitors (Mao et al., 1992; Mao et al., 1995a). Moreover, NMDA receptor antagonists prevent the development of antinociceptive tolerance to µ-opioid receptor agonists (Mao et al., 1998). Considering our previous finding that suppressed morphine antinociception against mechanical allodynia in the CFA-induced inflammatory pain state was partially restored by pretreatment with PKC inhibitor (Aoki et al., 2014b), blocking the NMDA receptors with a single treatment with methadone may prevent the phosphorylation of µ-opioid receptors by PKC in the CFA-induced inflammatory pain state.

## Conclusion

In conclusion, methadone is extremely effective against thermal hyperalgesia in the morphine-resistant inflammatory pain state. Moreover, a single injection of methadone restores suppressed morphine antinociception in the inflammatory pain state without altering the expression level of mRNA for µ-opioid receptors.

## Data Availability Statement

The raw data supporting the conclusions of this article will be made available by the authors, without undue reservation.

## Ethics Statement

The animal study was reviewed and approved by The Ethics Committee for Animal Experiments at Tohoku Medical and Pharmaceutical University.

## Author Contributions

HM organized the research and wrote the manuscript. CW, AK, and MY conducted the experiments. CW partially prepared manuscript. SS contributed for discussion.

## Funding

This work was supported by JSPS KAKENHI Grant Numbers 15K08678 and a MEXT (Ministry of Education, Culture, Sports, Science and Technology)-Supported Program for the Strategic Research Foundation at Private Universities (2015-2019).

## Conflict of Interest

The authors declare that the research was conducted in the absence of any commercial or financial relationships that could be construed as a potential conflict of interest.
